# Quantification of Neurotransmitters in Mouse Brain Tissue by Using Liquid Chromatography Coupled Electrospray Tandem Mass Spectrometry

**DOI:** 10.1155/2014/506870

**Published:** 2014-09-03

**Authors:** Tae-Hyun Kim, Juhee Choi, Hyung-Gun Kim, Hak Rim Kim

**Affiliations:** ^1^Department of Pharmacology, College of Medicine, Dankook University, Cheonan 330-714, Republic of Korea; ^2^Bioresources Regional Innovation Center, Soon Chun Hyang University, Asan 336-745, Republic of Korea; ^3^Translational Research Center, Institute of Bio-Science Technology, Dankook University, Cheonan 330-714, Republic of Korea

## Abstract

A simple and rapid liquid chromatography tandem mass spectrometry method has been developed for the determination of BH4, DA, 5-HT, NE, EP, Glu, and GABA in mouse brain using epsilon-acetamidocaproic acid and isotopically labeled neurotransmitters as internal standards. Proteins in the samples were precipitated by adding acetonitrile, and then the supernatants were separated by a Sepax Polar-Imidazole (2.1 mm × 100 mm, i.d., 3 *μ*m) column by adding a mixture of 10 mM ammonium formate in acetonitrile/water (75 : 25, v/v, 300 *μ*l/min) for BH4 and DA. To assay 5-HT, NE, EP, Glu, and GABA; a Luna 3 *μ* C_18_ (3.0 mm × 150 mm, i.d., 3 *μ*m) column was used by adding a mixture of 1% formic acid in acetonitrile/water (20 : 80, v/v, 350 *μ*l/min). The total chromatographic run time was 5.5 min. The method was validated for the analysis of samples. The calibration curve was linear between 10 and 2000 ng/g for BH4 (r^2^ = 0.995) , 10 and 5000 ng/g for DA (r^2^ = 0.997) , 20 and 10000 ng/g for 5-HT (r^2^ = 0.994) , NE (r^2^ = 0.993) , and EP (r^2^ = 0.993) , and 0.2 and 200 *μ*g/g for Glu (r^2^ = 0.996) and GABA (r^2^ = 0.999) in the mouse brain tissues. As stated above, LC-MS/MS results were obtained and established to be a useful tool for the quantitative analysis of BH4, DA, 5-HT, NE, EP, Glu, and GABA in the experimental rodent brain.

## 1. Introduction

Neurotransmitters (NTs) are signaling molecules, which play pivotal roles in neuronal communications in the central nervous system [1, 2]. It is reported that changes in NTs quantitation in several brain regions involve the development of many psychiatric diseases and neurodegenerative diseases [3, 4]. Generally, neurotransmitters are classified into two categories based on their chemical styles: (i) the small molecules (dopamine (DA), serotonin (5-HT), norepinephrine (NE), epinephrine (EP), glutamate (Glu), *γ*-aminobutyric acid (GABA), histamine, and endocannabinoids and (ii) the neuropeptides (enkephalin, endorphin, and substance P) [5].

The quantitation of various NTs as a small molecule in the brain, especially the aromatic monoamines, should be measured by using high-pressure liquid chromatography (HPLC) separation coupled with amperometric electrochemical detection (ECD). This method has been applied in NTs analysis over the last three decades [6–8]. However, it is still rather difficult to determine different types of NTs simultaneously in one sample owing to the limited capability of accommodating changes in the mobile phase composition. Another difficulty of incorporating this method is that the analytes can only be identified by a stable retention time matching [9]. Nevertheless, tandem mass spectrometry (MS/MS) can provide high specificity due to additional structure information and high sensitivity [10]. Therefore, it has been commonly used for the quantification of NTs in the brain by coupling with both gas chromatography (GC) and liquid chromatography (LC) [5, 11, 12]. Owing to various efficiencies and time consumption of derivatization, a simplified sample preparation using liquid chromatography coupled with electrospray tandem mass spectrometry (ESI-MS/MS) is widely employed to quantify the NTs and their metabolites in the brains without derivatization [5, 9, 13]. The use of isotope labeled internal standards is vital to the enhanced method performance because the isotope ratio measurements provide a measure of quality control for each analyte by compensating for changes in analyte, retention time, recovery, degradation, and changes in detector responses caused by coeluting contaminants [9].

In this study, we developed a sensitive, simple, and simultaneous method to quantify the six major NTs such as DA, 5-HT, NE, EP, Glu, and GABA in mouse brains [14, 15]. In addition, a tetrahydrobiopterin (BH4), a vital cofactor for the biosynthesis of the DA, 5-HT, and NE, was also measured in the same sample. To establish a novel method for the direct measurement of biologically active levels of BH4, DA, 5-HT, NE, EP, Glu, and GABA in the brain samples, the present study was performed using a high efficiency HILIC column for BH4 and DA, a Luna 3 *μ* C_18_ (3.0 mm × 150 mm, i.d., 3 *μ*m) column for 5-HT, NE, EP, Glu, and GABA with reversed-phase HPLC separation and an ESI-MS/MS, which could minimize the sample interferences. At the same time, the multiple reactions monitoring (MRM) scan mode was sensitive enough to identify and quantify the BH4 and NTs in this new method.

## 2. Materials and Methods

### 2.1. Materials

The (6R)-5,6,7,8-Tetrahydrobiopterin dihydrochloride, dopamine hydrochloride, serotonin hydrochloride, (−)-norepinephrine, (−)-Epinephrine, D-glutamic acid, and *γ*-aminobutyric acid were purchased from Sigma-Aldrich Corporation (St. Louis, MO, USA). Internal standards (IS) with isotope labeling were 2-(3,4-dihydorxyphenyl) ethyl-1,1,2,2-d_4_-amine HCl (dopamine-D_4_, 98% at %D); serotonin- *α*, *α*,*β*,*β*-d_4_ creatinine sulfate complex (serotonin-D_4_, 98% at %D); (±)-norepinephrine-2,5,6,*α*,*β*,*β*-d_6_ HCl (norepinepherine-D_6_, 98% at %D); (±)-Epinephrine-d_3_ (N-methyl-d_3_) (epinephrine-D_3_, 98% at %D);* L*-glutamic-2,3,3,4,4-d_5_ acid (glutamate-D_5_, 98% at %D); 4-aminobutyric-2,2,3,3,4,4-d_6_ acid (*γ*-aminobutyric acid-D_6_, 98% at %D). All the ISs were purchased from C/D/N ISOTOPES INC. (Pointe-Claire, Quebec, Canada). Epsilon-acetamidocaproic acid (AACA) was donated by Kuhnil pharmaceuticals (Seoul, Korea). Water was purified with a Milli-Q water purification system (Millipore, Bedford, MA, USA). All other chemicals and reagents were of analytical grade and used without further purification.

### 2.2. Determination of Biopterins, Neurotransmitters, and ISs from MS/MS

#### 2.2.1. BH4, BH2, Biopterin, and IS (AACA)

**Figure 1:**
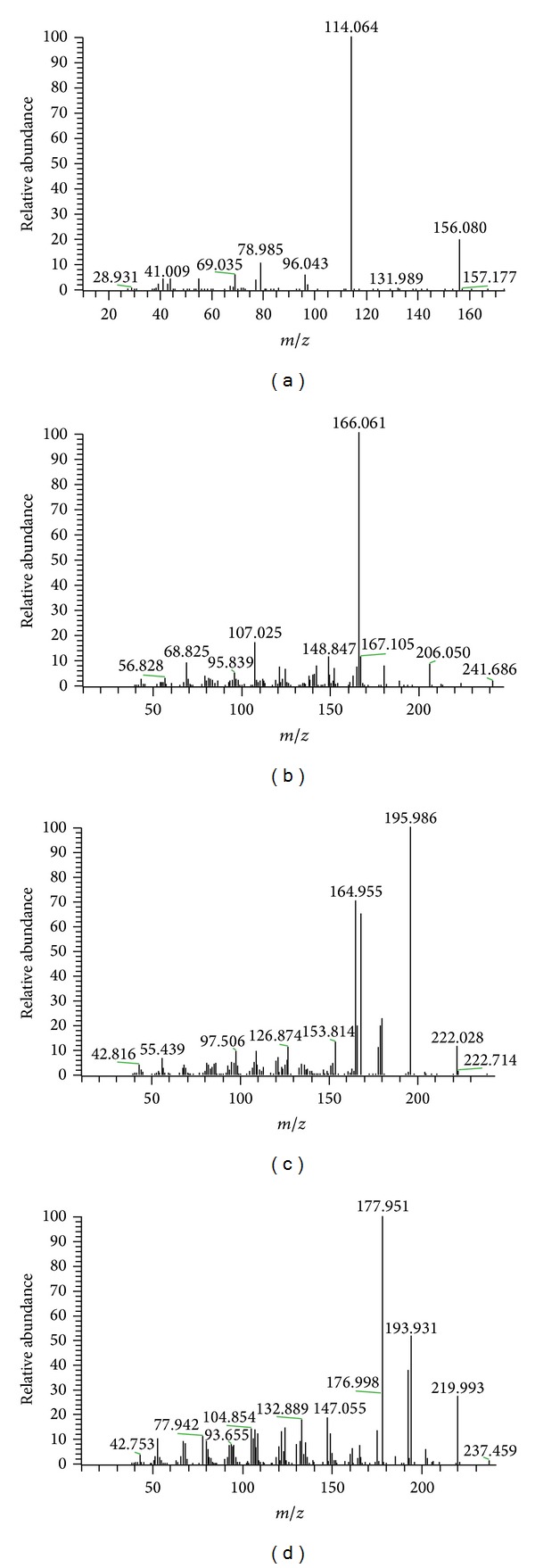
Product ion spectra for (a) epsilon-acetamidocaproic acid (AACA, precursor ion* m/z *174.1), (b) tetrahydrobiopterin (BH4, precursor ion* m/z *242.1), (c) dihydrobiopterin (BH2, precursor ion* m/z *240.0), and (d) biopterin (precursor ion* m/z *238.0).

Full-scan positive mass spectra of BH4 and the IS (AACA) reveal the protonated molecules, [M + H]^+^, of* m/z* 242.1 and 174, respectively. The mass-to-charge ratios of fragments of BH4 after fragmentation were 166, 107, and 149 and fragments of IS were 114, 156, and 79. The most abundant ion in the product ion spectra was 114 for IS (Figure 1(a)) and 166 for BH4 (Figure 1(b)). In parallel, full-scan positive mass spectra of BH2 and the biopterin showed the protonated molecules, [M + H]^+^, of* m/z* 240.0 and 238.0, respectively. The mass-to-charge ratios of fragments were 196.0, 164.9, and 168.0 in BH2 and of 177.9, 193.9, and 192.0 in biopterin. The most abundant ion in the product ion spectra was 196.0 for BH2 (Figure 1(c)) and 177.9 for biopterin (Figure 1(d)).

#### 2.2.2. Dopamine and Dopamine-D_4_ (IS)

**Figure 2:**
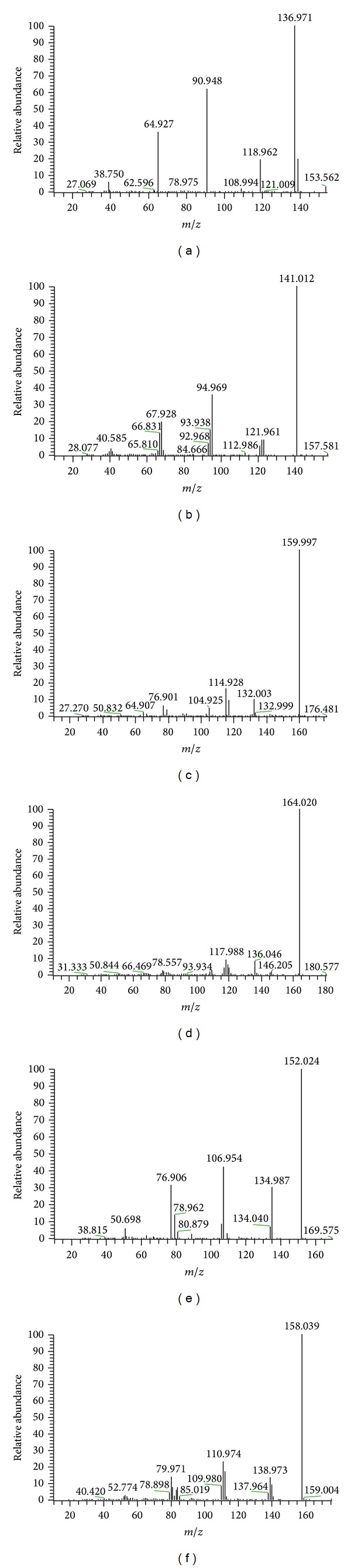
Product ion spectra for (a) dopamine, (b) dopamine-D_4_, (c) serotonin, (d) serotonin-D_4_, (e) norepinephrine, and (f) norepinephrine-D_6_.

Full-scan positive mass spectra of DA and the IS (dopamine-D_4_) showed that* m/z* of protonated molecules [M + H]^+^ are 154.1 and 158.1, respectively. The mass-to-charge ratios of fragments of DA after fragmentation were 137.0, 90.9, and 64.9 and of DA-D_4_ 141.0, 95.0, and 67.9. The most abundant ion in the product ion spectra was 137.0 for DA (Figure 2(a)) and 141.0 for IS (Figure 2(b)). But DA MRM had 154.1 to 90.9 due to matrix effects, which increased the mass-to-charge ratio level.

#### 2.2.3. Serotonin and Serotonin-D_4_ (IS)

Full-scan positive mass spectra of 5-HT and the ISs (serotonin-D_4_) showed that the mass-to-charge ratios of protonated molecules [M + H]^+^ were 177.0 and 181.0, respectively. After fragmentation, fragments of 5-HT seen were* m/z* 160.0, 114.9, and 132.0 and fragments of 5-HT-D_4_ *m/z* were 164.0, 118.0, and 136.0. The most abundant ion in the product ion spectra was at 160.0 for 5-HT (Figure 2(c)) and at 164.0 for IS (Figure 2(d)).

#### 2.2.4. Norepinephrine and Norepinephrine-D_6_ (IS)

Full-scan positive mass spectra of NE and the IS (norepinephrine-D_6_) showed that the mass-to-charge ratios of protonated molecules [M + H]^+^ were 170.1 and 176.1, respectively. After fragmentation, fragments of NE were* m/z* 152.0, 107.0, and 76.9 and fragments of NE-D_6_ *m/z* were 158.0, 111.0, and 112.0. The most abundant ion in the product ion spectra was at 152.0 for NE (Figure 2(e)) and at 158.0 for IS (Figure 2(f)). But* m/z* 170.1 to 107.0 and 176.1 to 111.0 was selected for NE and IS (NE-D_6_) MRM due to matrix effects, which increased the mass-to-charge ratio level.

#### 2.2.5. Epinephrine and Epinephrine-D_3_ (IS)

**Figure 3:**
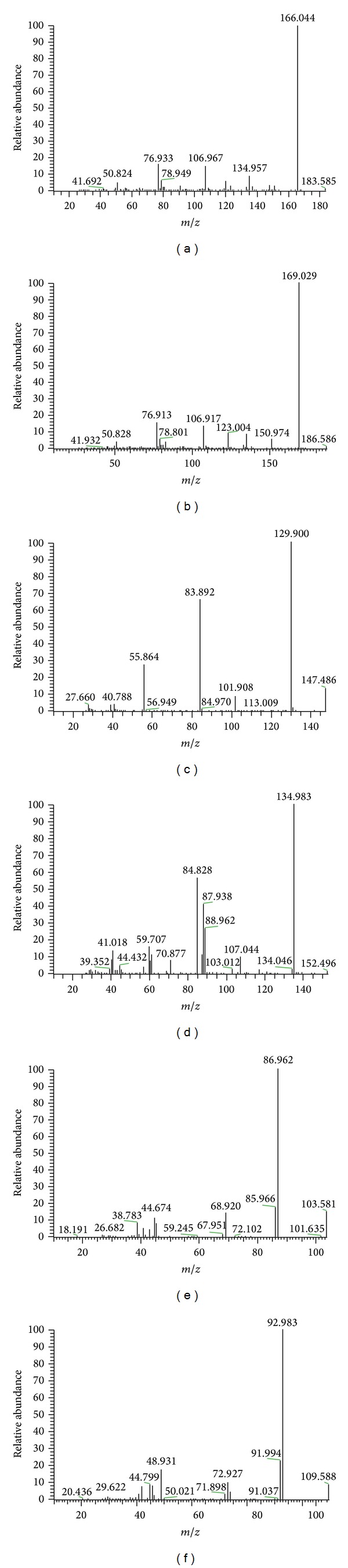
Product ion spectra for (a) epinephrine, (b) epinephrine-D_3_, (c) glutamate, (d) glutamate-D_5_, (e) *γ*-aminobutyric acid, and (f) *γ*-aminobutyric acid-D_6_.

Full-scan positive mass spectra of EP and the IS (epinephrine-D_3_) showed that the mass-to-charge ratios of protonated molecules [M + H]^+^ were 184.1 and 187.1, respectively. The* m/z *of fragments of EP after fragmentation were 166.0, 76.9, and 107.0 and of EP-D_3_ 169.0, 76.9, and 107.0, respectively. The most abundant ion in the product ion spectra was at 166.0 for EP (Figure 3(a)) and at 169.0 for IS (Figure 3(b)).

#### 2.2.6. Glutamate and Glutamate-D_5_ (IS)

Full-scan positive mass spectra of Glu and the IS (glutamate-D_5_) showed that the mass-to-charge ratios of protonated molecules [M + H]^+^ were 148.0 and 153.0, respectively. The mass-to-charge ratios of fragments of Glu after fragmentation were 129.0, 83.9, and 55.9 and of Glu-D_5_ were 135.0, 84.8, and 88.0, respectively. The most abundant ion in the product ion spectra was at 129.0 for glutamate (Figure 3(c)) and at 135.0 for IS (Figure 3(d)). But* m/z* 148.0 to 84.0 and* m/z* 153.0 to 88.0 for glutamate and IS (glutamate-D_5_) MRM were selected due to matrix effects, which increased the mass-to-charge ratio level.

#### 2.2.7. GABA and GABA-D_6_ (IS)

Full-scan positive mass spectra of GABA and the IS (GABA-D_6_) showed the protonated molecules, [M + H]^+^, of* m/z* 104.0 and 110.1, respectively. The mass-to-charge ratios of fragments of GABA after fragmentation were 87.0, 44.9, and 85.0 and of GABA-D_6_ were 93.0, 49.0, and 91.9, respectively. The most abundant ion in the product ion spectra was at 87.0 for GABA (Figure 3(e)) and at 93.0 for IS (Figure 3(f)).

### 2.3. Preparation of Stock Solution, Calibration Standards, and Quality Control Samples

Individual stock solution of each NT and isotope-labeled standard was prepared by accurate weighing of each compound (1 mg/mL methanol as the stock solution). The solution of BH4, DA, 5-HT, NE, EP, Glu, and GABA was prepared as a stock (1 mg/mL of each) with pure acetonitrile and then diluted with acetonitrile (50%) for each experiment. Standard solutions of BH4, DA, 5-HT, NE, EP, Glu, and GABA for calibration curves were prepared by spiking the blank solution prepared to the appropriate amounts, but added volumes were less than 10% of total DW volume. The final yielding concentrations for the standard curve were 10, 20, 50, 100, 200, 500, 1000, 2000, 5000, and 10000 ng/g for BH4 and dopamine. In parallel, the final concentrations for the standard curve were 20, 50, 100, 200, 500, 1000, 2000, 5000, and 10000 ng/g for 5-HT, NE, and EP. Likewise, the final concentrations for the standard curve were 0.2, 0.5, 1, 2, 5, 10, 20, 50, 100, and 200 *μ*g/g for Glu and GABA. The tolerance for reliable detection was 10 ng/g for BH4 and dopamine, 20 ng/g for 5-HT, NE and EP, and 0.2 *μ*g/g for Glu and GABA. The linear ranges and correlation coefficient of the calibration curve were summarized in Table 1. All the solutions were freshly prepared for each experiment.

### 2.4. Animals Care

ICR mice (male, body weight 20–30 g, *n* = 24), (Daehanbiolink Inc., Chungju, South Korea), were used. The mice were kept under a controlled condition (ambient temperature of 20 to 25°C, 12-h light/dark cycle). Food (Daehanbiolink Inc., Chungju, South Korea) and water were supplied* ad libitum*. NIH's guidelines for animal research were followed for all animal procedures and were approved by Institutional Animal Care and Use Committee (IACUC; DKU-12-018) which adheres to the guidelines issued by the Institution of Laboratory of Animal Resources (ILAR).

### 2.5. Sample Preparation of Specific Brain Regions

The specific brain regions of mouse were quickly dissected on an ice bath [16] and, subsequently, isolated brain tissues were homogenized with acetonitrile (1 mg/10 *μ*L) according to the internal standard (AACA: 100 ng/mL; dopamine-D_4_, serotonin-D_4_, norepinephrine-D_6_, epinephrine-D_3_, glutamate-D_5_, and GABA-D_6_: 1 *μ*g/mL). After a thorough homogenization, the BH4 and NTs (DA, 5-HT, NE, EP, Glu, and GABA) from brain tissues were extracted by sonication for 60s. The homogenates of brain tissue were centrifuged at 12,000 rpm for 10 min at 4°C. Supernatants were carefully transferred to 96-well plates and then injected onto the LC-MS/MS system by an autosampler for subsequent analysis. For determination of BH4 and NTs in mouse brain tissues, d-water was used as blank matrix.

### 2.6. Apparatus and Chromatographic Conditions

The liquid chromatographic system used was the Accela system (Thermo Fisher Scientific Inc., Waltham, MA, USA), equipped with a nanospace SI-2 3133 solvent delivery module as an autosampler (Shiseido Inc., Japan) and connected to Discovery Max (Thermo Fisher Scientific, Inc.) quadrupole tandem mass spectrometer coupled with electrospray ionization (ESI-MS/MS). System control and data analysis were performed using the Xcalibur software (Thermo Fisher Scientific, Inc.). Chromatographic separation was achieved using Hydrophilic Interaction Chromatography (HILIC) Sepax Polar-Imidazole (2.1 mm × 100 mm, i.d., 3 *μ*m particle size) HPLC column (Sepax Technologies, Delaware, USA) to assay BH4 and dopamine, and Luna 3 *μ* C18 (3.0 mm × 150 mm, i.d., 3 *μ*m particle size) to assay serotonin, norepinepherine, epinephrine, glautamte, and GABA with a Phenomenex C_18_ guard column (4 mm × 2 mm, Phenomenex). A nanospace SI-2 3004 column oven (Shiseido, Japan) was used online. To assay BH4 and dopamine, the mobile phase consisted of 10 mM ammonium formate (pH 3) in an acetonitrile/water (75 : 25, v/v) mixture. The flow rate was 300 *μ*L/min and the injection volume was 5 *μ*L. To assay 5-HT, NE, EP, Glu, and GABA, the mobile phase consisted of an acetonitrile/water (20 : 80, v/v) mixture. The flow rate was run at 350 *μ*L/min and the injection volume was 5 *μ*L. The electrospray ionization (ESI) mass spectrometer was operated in the positive ion mode. The optimal condition was as follows: the ESI needle spray voltage was 4000 V, the sheath gas pressure 35 unit, the auxiliary gas pressure 20 unit, the capillary temperature 206°C, the collision gas (Ar) pressure 1.5 mTorr, the skimmer offset 5 V, and the chrome filter peak width 10 s. Scanning was performed in profile mode with the SIM width 0.700 FWHM, scan time 0.200 s, and scan width 0.5 Da.

### 2.7. BH4 and NTs Assay Method Developed Using LC-MS/MS

**Figure 4:**
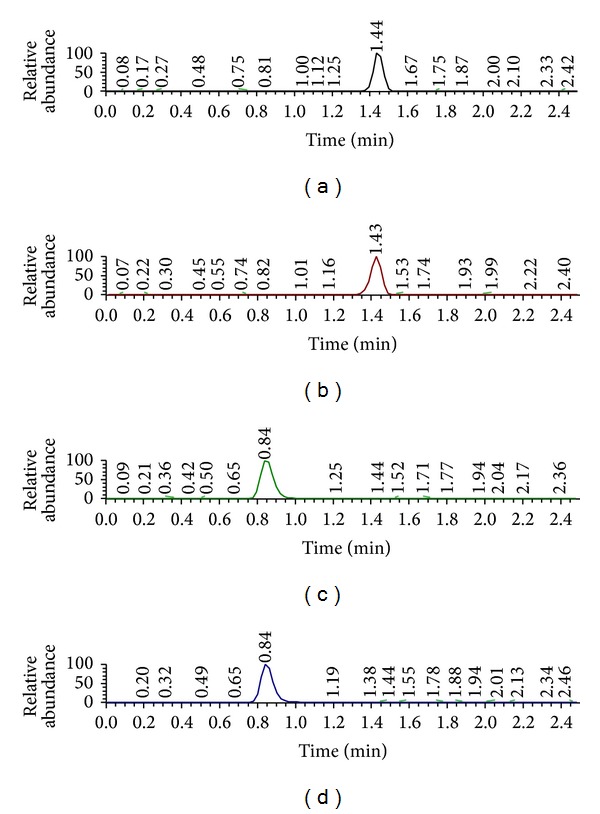
The LC-MS/MS chromatograms of BH4 (a), AACA (b), dopamine (c), and dopamine-D_4_ (d) using Polar-Imidazole (2.0 × 150 mm; i.d., 3 *μ*m) and mobile phase ACN: DW (75 : 25, v/v, 10 mM ammonium formate).

**Figure 5:**
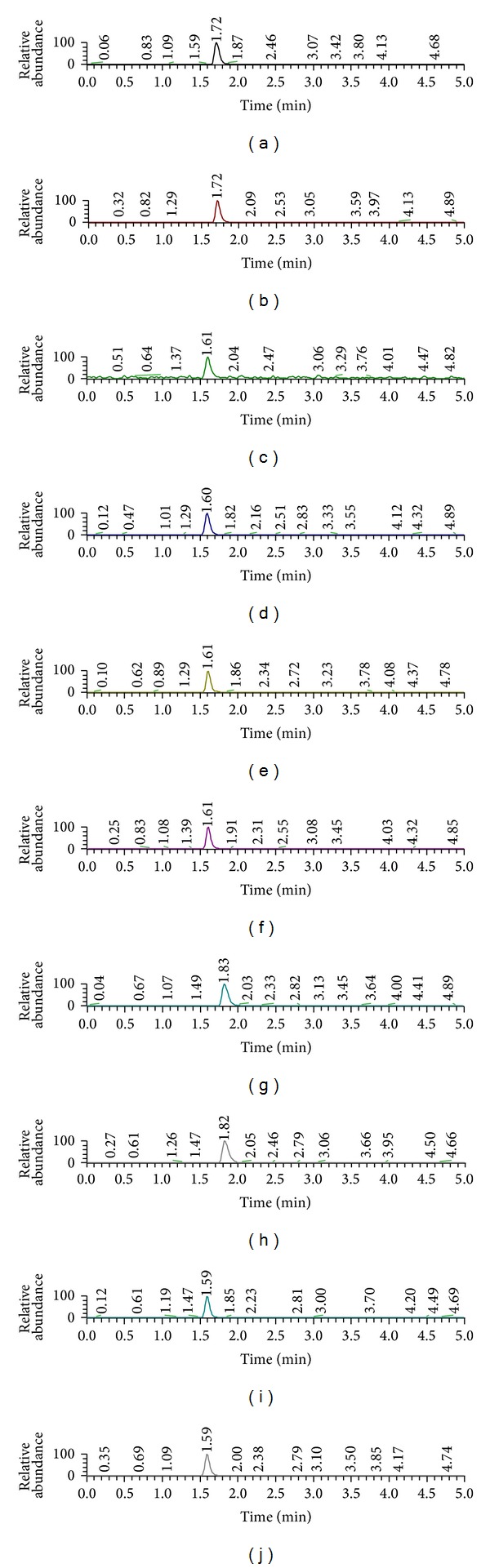
The LC-MS/MS chromatograms of neurotransmitters, (a) serotonin, (b) serotonin-D_4_, (c) norepinephrine, (d) norepinephrine-D_6_, (e) epinephrine, (f) epinephrine-D_3_, (g) glutamate, (h) glutamate-D_5_, (i) GABA, and (j) GABA-D_6_.

It was successful to qualify BH4 using Hydrophilic Interaction Chromatography (HILIC) Sepax Polar-Imidazole (2.1 mm × 100 mm, i.d., 3 *μ*m particle size) HPLC column (Sepax Technologies, Delaware, USA). The BH4 and IS Peak was settled in a matrix-free region. Moreover, the peaks had a symmetric shape, and we confirmed the LC-MS/MS chromatogram of BH4, AACA, DA, and DA-D_4_ (Figure 4). Following the same strategy, we analyzed successfully for 5-HT, NE, EP, Glu, and GABA by using Luna 3u C18 (3.0 mm × 150 mm, i.d., 3 *μ*m particle size). The peaks had a separated chromatogram and a symmetric shape (Figure 5).

### 2.8. Method Validation

The whole validation experiments followed the guideline of “FDA (US) [Guidance for Industry; Handling and Retention of BA and BE Testing Samples], May 2004.” To determine a linear range, eight nonzero calibration samples were employed. Linear regression of the ratio of peak area of BH4 or NTs to that of IS was done with weighting of 1/*X* ^2^ (least-squares linear regression analysis, where *X* is the concentration of the analyte). Precision and accuracy were evaluated by three different concentrations of QC solutions: interday precision was evaluated for 5 replicates per a single concentration. The value of accuracy was expressed as the mean of 25 replicates of determined concentration from 5 different analytical tests to the QC concentration (Table 2).

### 2.9. Statistical Analysis

All the values, tables, and figures given in the text are expressed as mean ± SD. Statistical differences between means were evaluated with two-tailed Student's *t*-test. *P* values less than 0.05 were taken to be statistically significant.

## 3. Results

### 3.1. Sample Preparation and Liquid Chromatography

For simple sample preparation, protein precipitation was attempted using acetonitrile. To prevent sample degradation and oxidation, an ascorbic acid with 0.01% (w/v) was also added and put in an ice bath. The peaks of BH4, dopamine, and IS were best when acetonitrile was used for protein precipitation and as an organic solvent of the mobile phase when using the HILIC column (Polar-Imidazole, 2.0 mm × 150 mm; i.d., 3 *μ*m) (Figure 4). Because BH4 and dopamine are easily dissolved in water, they are difficult to match the reverse column (C_18_) in chromatography analysis. However, HILIC column can match well with the hydrophilic chemicals. This study used a Polar-Imidazole column in analyzing BH4 and dopamine. The other NTs (5-HT, NE, EP, Glu, and GABA) were matched with a C18 column (Luna 3 *μ* C18 (3.0 mm × 150 mm, i.d., 3 *μ*m particle size)) (Figure 5), but the HILIC column could not separate peak of NTs clearly.

### 3.2. Mass Spectrometry of BH4, BH2, and Biopterin

The optimized electrospray ionization condition should be sensitive enough to detect BH4, DA, IS, BH2, and biopterin in positive ion detection mode. The most abundant protonated ion peaks ([M + H]^+^) in the Q1 mass spectra of BH4, DA, IS, BH2, and biopterin were at 242.1, 154.1, 174.0, 240.0, and 238.0, respectively (Figures 1 and 2). There was no evidence of fragmentation and adduct formation. The product ions in Q3 mass spectra and proposed fragmentation patterns were BH4, which becomes at 2-amino-7,8-dihydropteridin-4(1H)-one of* m/z* 166.0 by losing propane-1,2-diol. DA becomes butane-1,2-diol of* m/z* 90.9 by losing (E)-3-methylpent-3-en-1-amine; IS, (E)-*N*-ethylidenepentan-1-amine of* m/z* 114.0 by losing both carboxyl and hydroxyl groups; BH2, 2-amino-7,8-dihydro-6-(hydroxymethyl)pteridin-4(1H)-one) of* m/z* 196.0 by losing propan-2-ol; biopterin, 2-amino-6-(hydroxymethyl)pteridin-4(1H)-one) of* m/z* 196.0 by losing propan-2-ol. Also, to confirm separation between BH4 and other biopterins in biological samples, experiments were previously conducted to quantify biopterin, BH2, and BH4 in a mixed matrix [17].

### 3.3. Assay Optimization

The optimized electrospray ionization condition should be sensitive enough to detect BH4, DA, 5-HT, NE, EP, Glu, GABA, and ISs with positive ion detection mode. The most abundant protonated ion peaks ([M + H]^+^) in the Q1 mass spectra of BH4, DA, 5-HT, NE, EP, Glu, GABA, and ISs are listed in Table 3. There was no evidence of fragmentation and adduct formation. The product ions and collision energy in Q3 mass spectra of BH4, DA, 5-HT, NE, EP, Glu, GABA, and ISs were listed in Table 3.

### 3.4. Sensitivity and Specificity of BH4 and NTs in the Mouse Brain Tissue

Previously, our lab reported BH4 and dopamine levels in rat brain region [17]. To extend this method to mouse brain regions, we applied it the BH4 and NTs in the mouse brain. The standards for calibration were prepared by spiking them with DW. The peak areas of the spiked standard were constructed by subtracting the corresponding areas derived from the matrix. Meanwhile, calibration using internal standardization with deuterated analogues was performed. In biological specimen analysis, the isotope-labeled analogues of the targeted analyte are often recommended [9]. Due to their similar physicochemical properties, compared to deuterated analogues, the variability during sample preparation and ionization efficiency in the transfer of analytes from liquid to gas could be compensated for, and they could be differentiated ideally by their distinct mass-to-charge (*m/z*) ratios [18]. All analytes were subjected to HPLC-MS/MS analysis, and their distinct mass-to-charge (*m/z*) ratios were determined. The analytic parameters were listed in Table 1.

There are representative LC-MS/MS chromatograms of BH4, DA, and ISs (AACA and dopamine-D_6_) in the DW matrix (Figure 4). Also, there are 5-HT, NE, EP, Glu, GABA, and ISs LC-MS/MS chromatograms in the DW matrix (Figure 5). We tested the newly developed method using olfactory bulb (OB), frontal cortex (FC), hippocampus (HP), striatum (ST), hypothalamus (HT), pituitary gland (PT), midbrain (MB), cerebellum (CB), and brainstem (BS) from the mice and subsequently confirmed that the quantity of BH4 and NTs (Table 4).

#### 3.4.1. Linearity

Eight different concentrations from 10 to 2000 ng/g of BH4, from 10 to 5000 ng/g of DA, from 20 to 10000 ng/g of 5-HT, NE, and EP, and from 0.2 to 200 *μ*g/g of Glu, GABA is plotted against IS for the standard curves. This study establishes that the data from eight points are linear. The correlation coefficients (*r* ^2^), LOD, and LOQ of the standard curve are shown in Table 1.

### 3.5. Analysis of NTs in the Mice Brain Regions

The LC-MS/MS methodology was used to measure the levels of BH4 and NTs in nine brain regions including OB, FC, HP, ST, HT, PT, MB, CB, and BS from mice. The newly-developed LC-MS/MS method was used to analyze the quantity of BH4 and NTs in mice brain regions (Table 4). The endogenous levels of BH4, DA, 5-HT, NE, EP, Glu, and GABA were successfully detected and measured in mice brain regions.

## 4. Discussion

The present study was undertaken in order to describe a sensitive and specific LC-MS/MS method for simultaneous detection of BH4, DA, 5-HT, NE, EP, Glu, and GABA from mouse brain tissue. The principal advantages of using LC-MS/MS method include a simple purification procedure and a simple chromatographic condition using the MRM scan mode. The use of a HILIC column overcame the limitations of separating hydrophilic materials. Therefore, HILIC column could separate BH4 and DA from matrix effect with an appropriate retention time [19]. The other NTs (5-HT, NE, EP, Glu, and GABA) were matched well with a Luna 3 *μ* C18 column. The quantitative and confirmatory assurance comes from coeluting isotopically labeled internal standards [15]. So, the current developed method should be very useful for brain tissue works of research, regarding the analysis of the alternation of the levels of BH4, DA, 5-HT, NE, EP, Glu, and GABA.

This new method can enable measurement of BH4 and NTs rapidly and accurately in brain tissues. Previously, BH4 levels have been indirectly calculated by measuring the concentrations of biopterin in biological samples [18]. However the limitation of this indirect method is that it is unable to measure the exact BH4 levels owing to rapid oxidation and degradation. To avoid the problem, we tested several experimental conditions and found that a low temperature is a critical factor to prevent decomposition of BH4 in the brain tissues extract [17]. But the addition of antioxidant (DTE) and/or acid (HCl) to the samples does not affect dramatically the stability of BH4 [17]. Keeping the treated extracts at 4°C is necessary and enough to maintain BH4 stable for 4 hours, which is long enough to finish the analysis of the targets in samples. By using HILIC column, BH4 and DA were separated into single peaks. Under other methods, many unknown materials in the biological matrix interfered with the analysis of BH4 and DA in the biological samples [12]. But the use of a HILIC column could overcome the limitation to separating hydrophilic materials. So, HILIC column could separate successfully the BH4 and DA from matrix effect with an appropriate retention time (Figure 4). In addition, it could increase the sensitivity, selectivity, and accuracy of BH4 and DA in brain samples using MRM scan mode. Using Luna 3 *μ* C18 column (3.0 mm × 150 mm, i.d., 3 *μ*m particle size), 5-HT, NE, EP, Glu, and GABA were separated into single peaks. Also, the Luna 3 *μ* C18 column could separate NTs from matrix effect with an appropriate retention time, and the usage of MRM scan mode could increase the sensitivity, selectivity, and accuracy of NTs detection in brain samples (Figure 5).

The levels of BH4 and NTs were measured in several brain sections by using the newly-developed experimental method (Table 4). The BH4 is an essential cofactor for the aromatic acid hydroxylases, which are essential in the formation of NTs (DA, 5-HT, and NE), as well as for nitric oxide synthase (NOS), a vital enzyme for normal vascular and cardiac nitric oxide [20]. So, BH4 has been suggested to play a crucial role for many diseases. Therefore, it is necessary to reliably measure the biological concentration of BH4 for the evaluation of various diseases and for screening potential therapeutic candidates in neurological diseases [21]. These results showed that the BH4 level was at its highest in olfactory bulb, followed by cerebellum, frontal cortex, striatum, midbrain, pituitary gland, hypothalamus, brainstem, and hippocampus in a decreasing order. However, the DA level was at its highest in striatum, followed by hypothalamus, midbrain, pituitary gland, and olfactory bulb in a decreasing order. Interestingly, there were no detectable DA in hippocampus, frontal cortex, cerebellum, and brainstem. However, the lower limit of quantification in our method is 10 ng/g in samples. Therefore, even though there are some DA transmissions in these regions, the amount of DA in hippocampus, brain cortex, and brainstem could be below 10 ng/g. These data indicate that the level of BH4 could be distinctly correlated with the level of DA in the mouse brain tissue [17].

The 5-HT levels were at their highest in midbrain and brainstem, followed by hypothalamus, striatum, hippocampus, frontal cortex, occipital lobe, and cerebellum in a decreasing order. There was no detectable 5-HT in pituitary gland. The NE level was at its highest in hypothalamus and pituitary gland, followed by brainstem, midbrain, olfactory blub, cerebellum, striatum, and frontal cortex in a decreasing order. The EP level was at its highest in hippocampus, followed by hypothalamus, brainstem, midbrain, olfactory blub, frontal cortex, and pituitary gland in a decreasing order. However, there was no detectable EP in cerebellum; the levels of NE and EP have similar order in mouse brain sections. The Glu level was at its highest in hippocampus, followed by frontal cortex, striatum, hypothalamus, cerebellum, midbrain, brainstem, olfactory blub, and pituitary gland in a decreasing order. The GABA level was at its highest in hypothalamus, olfactory bulb, and hippocampus, followed by midbrain, frontal cortex, striatum, cerebellum, striatum, and brainstem in a decreasing order. Interestingly, the levels of Glu and GABA were detected as microgram based units, but others were detected as nanogram based units. These results suggested that the neurotransmitters in mouse brain were differentially released to do their function in brain sections.

However, a simple and rapid liquid chromatography tandem mass spectrometry (LC-MS/MS) method has been developed for the determination of BH4, DA, 5-HT, NE, EP, Glu, and GABA in mouse brain; the quantitative determination of endogenous neurotransmitters in brain regions by chromatographic coupled mass spectrometry presented here still has a limitation because of the typical lack of analyte-free matrix. There is no analyte-free sample of the authentic matrix; therefore, we have to use a surrogate matrix containing the authentic analyte [22].

## 5. Conclusions

A simple and rapid liquid chromatography tandem mass spectrometry (LC-MS/MS) method has been developed for the determination of BH4, DA, 5-HT, NE, EP, Glu, and GABA in mouse brain using epsilon-acetamidocaproic acid (AACA) and isotopically labeled neurotransmitters as an internal standard. Although it is clear that further studies are necessary to understand the physiological meaning of the different levels of BH4 and NTs, this new method could be applied for tracking the changes of the endogenous BH4 and NTs which are affected significantly by various stimuli or in neurodegenerative diseases [10, 23].

## Figures and Tables

**Table 1:** The calibration of neurotransmitters by LC-MS/MS.

Chemical	Internal standard	Equations^a^	Linear range (ng/g)	Correlation coefficient (*R* ^2^)	LOD (ng/g)	LOQ (ng/g)
BH4	AACA	*y* = 2.89 × 10^−5^ *x* − 2.8 10^−4^	10–10000	0.9964	1	10
Dopamine	Dopamine-D_4_	*y* = 5.03 × 10^−5^ *x* − 2.310^−5^	10–10000	0.9967	1	10
Serotonin	Serotonin-D_4_	*y* = 9.92 × 10^−5^ *x* − 1.69 10^−4^	20–10000	0.9940	2	20
Norepinephrine	Norepinephrine-D_6_	*y* = 1.20 × 10^−4^ *x* + 9.3 10^−3^	20–10000	0.9927	2	20
Epinephrine	Epinephrine-D_3_	*y* = 8.00 × 10^−5^ *x* + 2.86 10^−4^	20–10000	0.9929	2	20
Glutamate	Glutamate-D_5_	*y* = 0.1409*x* + 1.16 10^−2^	200–200,000	0.9964	20	200
GABA	GABA-D_6_	*y* = 0.0453*x* − 1.57 10^−4^	200–200,000	0.9986	20	200

LOD; limit of detection. LOQ; limit of quantitation.

^a^The calibration curves were constructed by plotting the peak area ratio to IS versus the concentration of each analyte.

**Table 2:** Determination of neurotransmitters by LC-MS/MS: validation results on precision and accuracy.

Chemical	Intraday precision^a^			Interday precision^b^			Accuracy (%)			RE (%)		
	Low	Mid	High	Low	Mid	High	Low	Mid	High	Low	Mid	High
BH4	1.18	1.09	0.42	4.28	1.78	0.59	102.90	99.72	97.48	2.90	0.28	2.53
Dopamine	1.15	1.26	0.73	3.98	1.90	0.86	103.07	99.12	97.80	3.07	0.88	2.21
Serotonin	8.30	8.12	8.20	8.58	9.44	9.23	95.57	108.46	96.15	4.43	8.46	3.85
Norepinephrine	8.21	6.95	4.50	10.17	7.28	4.93	104.90	103.06	98.75	4.90	3.06	1.26
Epinephrine	6.22	4.51	2.45	6.88	7.50	2.57	93.00	93.90	97.91	7.00	6.10	2.09
Glutamate	3.13	4.18	3.16	3.63	4.26	3.41	101.00	97.10	95.81	1.00	2.90	4.19
GABA	2.76	3.77	3.47	3.70	4.15	4.06	103.50	101.27	93.94	3.50	1.27	6.06

For BH4, Dopamine, Serotonin, norepinephrine, epinephrine, the low, medium, and high control solutions were 30 pg/mg, 500 pg/mg, and 8000 pg/mg of DW, respectively. For glutamate and GABA, those were 6000 pg/mg, 30,000 pg/mg, and 160,000 pg/mg of DW.

For both precision tests, the values were in coefficient of variation (CV).

RE = Relative Error.

^a^Mean of five replicates (*n* = 5) observations at each concentration.

^b^Mean of 25 replicates (*n* = 25) observations over five different analytical runs.

**Table 3:** The analytic parameters of neurotransmitters by LC-MS/MS.

Chemical	Precursor Ion (*m*/*z*)^a^	Collision energy^b^	Product ion^c^	Retention time (min)
BH4	242.1	20	166.0	1.44
AACA	174.0	14	114.0	1.43
Dopamine	154.1	24	90.9	0.84
Dopamine-D_4_	158.1	9	141.0	0.84
Serotonin	177.0	10	160.0	1.72
Serotonin-D_4_	181.0	12	164.0	1.72
Norepinephrine	170.1	20	107.0	1.61
Norepinephrine-D_6_	176.1	20	111.0	1.60
Epinephrine	184.1	9	166.0	1.61
Epinephrine-D_3_	187.1	9	169.0	1.61
Glutamate	148.0	16	83.9	1.83
Glutamate-D_5_	153.0	16	88.0	1.82
GABA	104.0	10	86.9	1.59
GABA-D_6_	110.1	10	93.0	1.59

GABA, *γ*-aminobutyric acid.

^a^The detected chemicals had the greatest responses under the positive mode: the [M + H]^+^ was used as the precursor ion.

^b^The collision energy was optimized to have the greatest product ion intensity.

^c^The product ion was used for the MRM analysis.

**Table 4:** The levels of tetrahydrobiopterin (BH4) and neurotransmitters in mouse brain regions.

Brain regions	BH4 (ng/g)	DA (ng/g)	5-HT (ng/g)	NE (ng/g)	EP (ng/g)	Glu (*μ*g/g)	GABA (*μ*g/g)
Striatum	164.0 ± 21.79	3463 ± 200.9	207.7 ± 17.81	457.6 ± 73.59	28.55 ± 3.06	1037 ± 86.57	397.4 ± 7.466
Midbrain	129.2 ± 9.86	59.8 ± 6.19	486.7 ± 50.10	802.5 ± 60.52	73.5 ± 9.54	670.2 ± 98.11	682.2 ± 34.66
Hippocampus	21.5 ± 1.40	ND	98.1 ± 13.64	3170 ± 669.1	281.3 ± 33.37	3507 ± 1431	721.0 ± 56.15
Olfactory bulb	577.2 ± 59.79	40.6 ± 7.34	84.93 ± 9.88	734.4 ± 95.53	59.7 ± 14.59	597.8 ± 90.36	772.1 ± 92.21
Frontal cortex	212.5 ± 52.27	ND	81.83 ± 6.25	320.1 ± 55.30	44.9 ± 6.85	1090 ± 108.1	430.8 ± 17.47
Hypothalamus	84.8 ± 14.17	111.9 ± 26.17	282.4 ± 28.42	2627 ± 135.4	188.0 ± 27.68	811.8 ± 136.8	829.7 ± 65.56
Cerebellum	293.4 ± 69.74	ND	29.17 ± 4.07	584.5 ± 79.12	ND	791.9 ± 62.29	389.9 ± 14.88
Brainstem	60.9 ± 9.02	ND	452.5 ± 50.03	1123 ± 82.11	78.4 ± 9.71	622.5 ± 37.00	348.9 ± 18.10
Pituitary gland	92.2 ± 19.29	48.9 ± 18.9	ND	2539 ± 358.5	35.5 ± 9.00	536.6 ± 70.25	22.03 ± 1.733

Unit: Mean ± SEM ug/tissue weight (g).
